# *Sarcocystis nesbitti* Causes Acute, Relapsing Febrile Myositis with a High Attack Rate: Description of a Large Outbreak of Muscular Sarcocystosis in Pangkor Island, Malaysia, 2012

**DOI:** 10.1371/journal.pntd.0002876

**Published:** 2014-05-22

**Authors:** Claire M. Italiano, Kum Thong Wong, Sazaly AbuBakar, Yee Ling Lau, Norlisah Ramli, Sharifah Faridah Syed Omar, Maria Kahar Bador, Chong Tin Tan

**Affiliations:** 1 Department of Medicine, Faculty of Medicine, University of Malaya, Kuala Lumpur, Malaysia; 2 Department of Pathology, Faculty of Medicine, University of Malaya, Kuala Lumpur, Malaysia; 3 TIDREC, Department of Medical Microbiology, Faculty of Medicine, University of Malaya, Kuala Lumpur, Malaysia; 4 Department of Parasitology, Faculty of Medicine, University of Malaya, Kuala Lumpur, Malaysia; 5 Department of Medical Imaging, Faculty of Medicine, University of Malaya, Kuala Lumpur, Malaysia; 6 Department of Medical Microbiology, Faculty of Medicine, University of Malaya, Kuala Lumpur, Malaysia; University of California San Diego School of Medicine, United States of America

## Abstract

**Background:**

From the 17^th^ to 19^th^ January 2012, a group of 92 college students and teachers attended a retreat in a hotel located on Pangkor Island, off the west coast of Peninsular Malaysia. Following the onset of symptoms in many participants who presented to our institute, an investigation was undertaken which ultimately identified *Sarcocystis nesbitti* as the cause of this outbreak.

**Methodology/Principal Findings:**

All retreat participants were identified, and clinical and epidemiological information was obtained via clinical review and self-reported answers to a structured questionnaire. Laboratory, imaging and muscle biopsy results were evaluated and possible sources of exposure, in particular water supply, were investigated. At an average of 9–11 days upon return from the retreat, 89 (97%) of the participants became ill. A vast majority of 94% had fever with 57% of these persons experiencing relapsing fever. Myalgia was present in 91% of patients. Facial swelling from myositis of jaw muscles occurred in 9 (10%) patients. The median duration of symptoms was 17 days (IQR 7 to 30 days; range 3 to 112). Out of 4 muscle biopsies, sarcocysts were identified in 3. *S. nesbitti* was identified by PCR in 3 of the 4 biopsies including one biopsy without observed sarcocyst. Non-Malaysians had a median duration of symptoms longer than that of Malaysians (27.5 days vs. 14 days, p = 0.001) and were more likely to experience moderate or severe myalgia compared to mild myalgia (83.3% vs. 40.0%, p = 0.002).

**Conclusions/Significance:**

The similarity of the symptoms and clustered time of onset suggests that all affected persons had muscular sarcocystosis. This is the largest human outbreak of sarcocystosis ever reported, with the specific *Sarcocystis* species identified. The largely non-specific clinical features of this illness suggest that *S. nesbitti* may be an under diagnosed infection in the tropics.

## Author Summary

*Sarcocystis* species are protozoan organisms that have been associated with disease in animals but less frequently so in humans. Following a retreat on Pangkor Island off Peninsular Malaysia, a number of persons presented to our hospital with prolonged fever and muscle pain that was initially difficult to attribute to a known infectious cause. Investigations, including muscle biopsies and PCR, showed that this outbreak was most likely due to *Sarcocystis nesbitti* infection. The most common clinical features were fever and myalgia that was relapsing-remitting in more than half the patients. Some patients had visible swelling of muscle groups, including of the face, with magnetic resonance imaging also demonstrating inflammation in these muscles. Herein, we present the clinical and investigation findings in 89 symptomatic persons in the largest reported outbreak of human muscular sarcocystosis to date. Our findings provide insights and suggestions for the most appropriate forms of investigation, treatment and possible source of infection.

## Introduction

*Sarcocystis* spp. are intracellular protozoan parasites which may involve humans either as definitive or intermediate hosts. Humans are definitive hosts for *Sarcocystis hominis* and *Sarcocystis suihominis*, acquired by consuming undercooked sarcocyst-containing beef or pork, respectively. Humans can also become accidental intermediate hosts for other *Sarcocysti*s species by consuming food or water contaminated with fecal sporocysts from an infected definitive host. In such cases, hematogenous dissemination can occur with invasion of muscle leading to sarcocysts [1].

As a disease, sarcocystosis is noted in a variety of animals [2] but symptomatic human disease appears to be less common, with fewer than 150 cases reported in the literature [1], [3], [4]. The prevalence of incidental sarcocysts in humans is also difficult to establish. A previous report showed a series of autopsy tongue muscle collected in the University of Malaya Medical Centre (UMMC), Malaysia, to be positive for sarcocysts in 21% of cases [5], yet, of the more than 1,500 limb muscle biopsies received in the past 20 years in the same centre for routine diagnosis of various symptomatic muscle diseases, none have yielded any sarcocyst-positive tissue (Wong KT, unpublished data).

The largest clustered outbreak of symptomatic muscular sarcocystosis previously reported was in 6 American military servicemen involved in a Malaysian jungle mission [3]. There were also recent reports involving a total of 100 foreign persons with sporadic acute muscular *Sarcocystis*-like illness after returning from Tioman Island, off the east coast of Peninsular Malaysia, between 2011 and 2012 [4]. The association of *S. nesbitti* infection with symptomatic human sarcocystosis has also been recognized recently [6], [7]. Herein, we report symptomatic muscular sarcocystosis affecting 89 of 92 persons following a retreat in January 2012 in Pangkor Island, off the west coast of Peninsular Malaysia. This report adds to previous molecular work [6], [7] and limited clinical studies by providing a comprehensive overview of the clinical features and time course of the illness. Furthermore, the role and results of blood and imaging investigations, and muscle biopsy, in influencing diagnosis and a consideration of management options is presented.

## Methods

### Case Definition and Outbreak Investigation

A case was defined as a person who attended a specified retreat at a hotel on Pangkor Island, Malaysia, from the 17^th^ to 19^th^ January 2012, and developed relevant clinical symptoms (fever, headache, myalgia and/or arthralgia) within 28 days upon return. Cases were subsequently defined as ‘definite sarcocystosis’ if there was histological demonstration or nucleotide sequences of *Sarcocystis* spp. from muscle tissue. The remaining cases were defined as ‘probable sarcocystosis’. All persons who attended the retreat, whether or not they had medical reviews at UMMC, submitted self-reported responses to a structured questionnaire aimed at ensuring that all cases were identified. To further elucidate the clinical features, participants were asked about the duration of symptoms, episodes of relapse and to describe the severity of myalgia. Myalgia was defined as ‘severe’, if “excruciating” pain was experienced, ‘moderate’, if daily activities were affected and analgesics required, and ‘mild’ if daily activities were not affected and analgesics not required. Fever referred to a subjective sensation of fever as reported by the patient. If there was any conflicting information between the initial medical review and questionnaire responses, the medical review was taken as more accurate. Participants were also questioned regarding activities and food or water exposure to ascertain potential exposure risks during the retreat. They were also asked if any family members or contacts who did not attend the retreat reported similar symptoms.

To investigate possible sources of infection, water samples were examined as previously described [8]. Ten litre samples were collected from different places along the “gravity-feed” water supply system (up-, middle, down stream of the water source, and from water tanks in the hotel) approximately 3 months after the outbreak.

### Investigations

Investigations included full blood counts (FBC), renal function tests (RFT), liver function tests (LFT), serum creatine kinase (CK) levels, chest x-rays, blood cultures, and blood films for malarial parasites. Results were considered ‘early’ if obtained before 12^th^ February 2012 (less than 4 weeks after the start of the retreat) or ‘late’ if obtained after.

Serological testing was done for chikungunya and dengue viruses, *Legionella*, *Mycoplasma*, and *Leptospira*. Testing was performed using an immunofluorescence assay (in-house) for detection of chikungunya IgM and IgG; anti-dengue IgM and IgG capture ELISA (Standard Diagnostics, Inc, Korea) for detection of dengue IgM and IgG; immunofluorescence assays for detection of *Legionella* IgG (MarDx Diagnostics, Inc, Ireland) and *Legionella* IgM (Vircell S.L., Spain); SERODIA-MYCO II (Fujirebi Inc., Japan) for detection of *Mycoplasma* total antibodies, and microscopic agglutination test (in-house) for detection of *Leptospira* total antibodies. *Sarcocystis* serology was done at the CDC by an immunoblot assay using *S.neurona* merozoite-derived antigens (personal communication, CDC, Atlanta, USA) in 10 patients.

Magnetic resonance imaging (MRI) of skeletal muscles was performed in 8 patients who underwent whole-body coronal T2 weighted, T1 weighted and T2 weighted short inversion time inversion recovery (STIR) scans using the 1.5 T SignaHDx MR Systems (GE Healthcare, USA).

Muscle biopsies from affected sites in 4 patients with myalgia and MRI abnormalities were fixed in buffered 10% formalin and routinely processed. Hematoxylin and eosin stained tissue sections were examined by light microscopy. Polymerase chain reaction (PCR) for *Sarcocystis* spp. was performed on all 4 biopsies (6,7).

### Statistical Analysis

Statistical analysis was performed using chi-square testing and Fisher's exact test to compare the categorical variables in relation to patients' nationality and sex. The Mann-Whitney U-test was utilized to compare the median duration of symptoms between nationalities and sexes while Spearman's rank correlation and independent samples t-test were used to analyze the relationship between age and severity of myalgia, and duration of symptoms, respectively.

IBM SPSS Statistics version 21.0 (Armonk, NY: IBM Corp) was used for statistical analysis. P values<0.05 were considered significant.

### Ethics Statement

**Figure 1:**
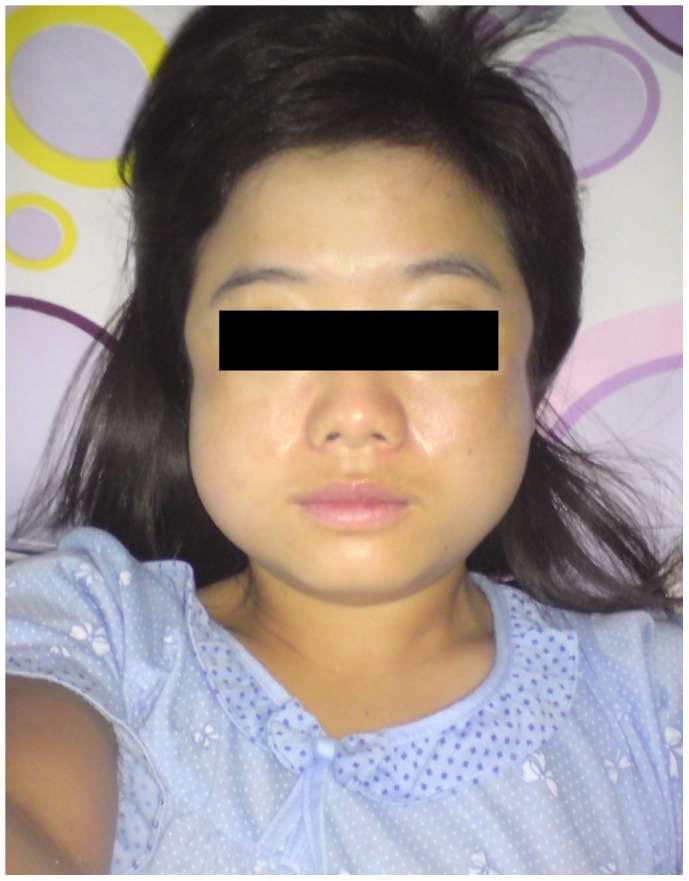
32 year old student from China with facial swelling from swollen temporalis and masseter muscles (Case 1, Table 3).

This investigation was undertaken in response to the presentation of acutely ill patients to our institution with the intent of determining the infectious agent responsible for the outbreak. Patients and their guardians were informed throughout their management that investigations were undertaken to ensure that critical illness did not develop in any person, to identify the causative organism, and, if possible, to determine the mode of acquisition of infection and henceforth, to prevent further infection. Ethics or IRB approval was not requested for this outbreak investigation. All patients who underwent muscle biopsy, the only invasive test, gave written informed consent. Written consent was obtained from the patient in Figure 1 for publication of the photograph.

## Results

### Clinical Presentation and Features

From 17^th^ to 19^th^ January 2012, a group of 92 college students and teachers attended a retreat at a hotel located on Pangkor Island, Malaysia. There were 55 males and 37 females, with a median age of 35 years (IQR 27 to 44; range, 5 to 64). Seventy-one were Malaysians and 21 non-Malaysians. A total of 58 cases were medically assessed at our institute, and 9 were admitted to hospital.

From a review of available medical records and questionnaire responses, it was determined that 89 persons demonstrated or reported features consistent with the case definition. Of these, 53 were male and 36 female with a median age of 34 years (IQR 27 to 43.5). Sixty-nine (77.5%) symptomatic persons were Malaysians and 20 (22.5%) were non-Malaysians: 9 from China, 3 from Nepal, 2 each from Korea and Indonesia and 1 person each from India, Netherlands, Myanmar and Iran, respectively. Malaysians were older with a median age of 38 years (IQR 27.50 to 45.00) compared to 29·5 years (IQR 24.25 to 33.75) for non-Malaysians (p = 0.012).

The onset of symptoms could be accurately ascertained for 82 patients and occurred between day 1 and day 26 post-retreat. Symptoms began between day 9 and 11 for 58 (70.7%) of these patients. The most common symptoms and their frequencies are listed in Table 1. Retreat participants did not report any similar illness in colleagues or family members who did not attend the retreat.

**Table 1:** Frequency of symptoms, duration and site of myalgia reported by patients (n = 89).

Symptom	Location/duration of myalgia	Number of patients with symptom/Total number of patients reporting or affected patients (%)
Fever		84/89 (94.4)
Relapsing fever		48/84 (57.1)
Myalgia	Any muscle group	81/89 (91.0)
	Lower limbs	57/81 (70.3)
	Back	49/81 (60.5)
	Upper limbs	46/81 (56.8)
	Neck	34/81 (42.0)
	Face/jaw	5/81 (6.2)
Duration of myalgia	<1 week	21/81 (25.9)
	1 to <2 weeks	16/81 (19.7)
	2 to <4 weeks	17/81 (21.0)
	4 to <8 weeks	22/81 (27.2)
	>8 weeks	5/81 (6.2)
Headache		77/89 (86.5)
Cough		36/89 (40.4)
Joint Pain		35/89 (39.3)
Nausea		25/89 (28.1)
Vomiting		16/89 (18.0)
Diarrhoea		16/89 (18.0)
Rash		4/89 (4.5)

Overall, the total duration of symptoms lasted from 3 days to nearly 4 months with a median duration of 17 days (IQR 7 to 30). Twenty-seven patients (30.3%) experienced symptoms for 1 month or longer, myalgia being the most prolonged symptom. There was no association between age and duration of symptoms, and no significant difference in median age between the sexes. For non-Malaysians, the median duration of symptoms was 27.5 days (IQR, 17.3 to 42.0) which was significantly longer than the 14 days (IQR, 7.0 to 29.5) experienced by Malaysians (p = 0.001).

In the “early” phase of illness, myalgia was particularly marked in the neck muscles. Later it was experienced most commonly in the lower limbs followed by the back and the upper limbs. Among the 73 persons who graded their myalgia for severity, severe myalgia was reported by 10 persons (13·7%), moderate myalgia by 27 (37·0%), and mild myalgia by 36 (49.3%). There was no significant difference in the median age of those with mild or moderate/severe pain and no significant difference between the sexes. Sixty-two (89.9%) Malaysians and 19 (95.0%) non-Malaysians reported myalgia as a symptom but this was not significantly different. However, 15 out of 18 (83.3%) non-Malaysians reported myalgia as moderate/severe compared to 22 out of 55 (40.0%) Malaysians (p = 0.002). Thus, non-Malaysians were more likely than Malaysians to experience moderate/severe myalgia.

Relapsing fever was reported in 48 of the 84 (57.1%) patients with fever, and 15 (31·3%) of the relapsing fever cases had 3 or more cycles of fever. Each symptomatic episode lasted a median of 5 days (IQR, 3 to 7; range, 1 to 21) and each remission 4 days (IQR 3 to 7; range, 1 to 30). There was no particular pattern regarding the duration of the first or subsequent cycles. Of the 9 patients admitted to hospital, 8 recorded temperatures greater than or equal to 39°C.

Between 34 and 38 days post-retreat, 9 patients developed visible facial muscle swelling with resolution in all patients within 7 days (Figure 1). One of these patients also reported swelling of the thenar eminence of one hand. Four patients presented with calf muscle swelling and another patient had isolated swelling of the interossei muscles of one hand. The higher proportion of non-Malaysians with muscle swelling (5 cases; 25.0%) compared to non-Malaysians (9 cases; 13.0%) was not statistically significant.

Cough was reported as non-productive, transient, and seen only in the first 2 weeks of illness.

### Microbiology

Blood cultures from 21 patients were negative for bacteria or fungal growth. Initial chikungunya IgM serology using immunofluorescent staining (IFAT) was positive in 53% (43/81) of patients. However, chikungunya IgG serology up to two weeks after onset of illness confirmed seroconversion in only one patient.

Ten paired patient sera were serologically tested for sarcocystosis including 3 ‘definite sarcocystosis’ cases. There were 3 positive results in both acute and convalescent sera, 2 seroconverted positives, 1 equivocal, and 4 cases that were negative for both acute and convalescent samples. In five patients who had muscle swelling, 3 had positive results. One of the definite cases of sarcocystosis had negative serology.

None of the other serology tests for dengue, *Legionella*, *Mycoplasma* or *Leptospira* were consistent with recent infection.

### Blood Test

FBC, LFTs, and CK abnormalities varied according to the time-point of illness. Those parameters that were most commonly abnormal ‘early’ or ‘late’ in the course of the infection are shown in Table 2. Only those symptomatic patients who were reviewed in the hospital had blood tests performed in the early period.

**Table 2:** Peripheral blood parameter results in early and late disease.

Peripheral blood parameter	Time point	Number of patients with elevated parameter/number of patients tested (%)	Median (×10^9^ or IU/L or U/L)	IQR (×10^9^ or IU/L or U/L)	Number of patients with both early and late bloods/changes with time
Eosinophil count (0.02–0.50×10^9^/L)	Early	18/57 (31.6)	0.37	0.20–0.54	12/1 elevated early and late, 7 normal early and elevated late, 4 normal early and late
	Late	10/15 (66.7)	0.79	0.46–0.99	
Lymphocyte count (1.0–3.0×10^9^/L)	Early	19/59 (32.2)	2.40	1.75–3.39	12/1 elevated early and late, 6 normal early and elevated late, 4 normal early and late, 2 elevated early and normal late
	Late	9/15 (60.0)	3.21	2.30–4.70	
Alanine aminotransferase (30–65 IU/L)	Early	36/56 (64.3)	86.00	53.00–145.00	25/10 elevated early and late, 1 normal early and elevated late, 4 normal early and late, 10 elevated early and normal late
	Late	12/27 (44.4)	63.00	47.00–95.00	
Aspartate aminotransferase (15–37 IU/L)	Early	39/56 (69.6)	46.00	33.50–62.75	25/12 elevated early and late, 2 normal early and elevated late, 2 normal early and late, 9 elevated early and normal late
	Late	16/27 (59.3)	38.00	29.00–52.00	
γ-glutamyltransferase (5–55 IU/L)	Early	35/56 (62.5)	70.00	39.00–143.25	25/12 elevated early and late, 1 normal early and elevated late, 5 normal early and late, 7 elevated early and normal late
	Late	15/27 (55.6)	61.00	39.00–106.00	
Creatine Kinase (26–192 U/L)	Early	7/48 (14.6)	95.00	64.5–151.50	7/1 elevated early and late, 5 normal early and elevated late, 1 normal early and late
	Late	9/10 (90.0)	921.00	400–1489.00	

Early – <4 weeks after start of retreat. Late – >4 weeks after start of retreat.

### Magnetic Resonance Imaging

**Figure 2:**
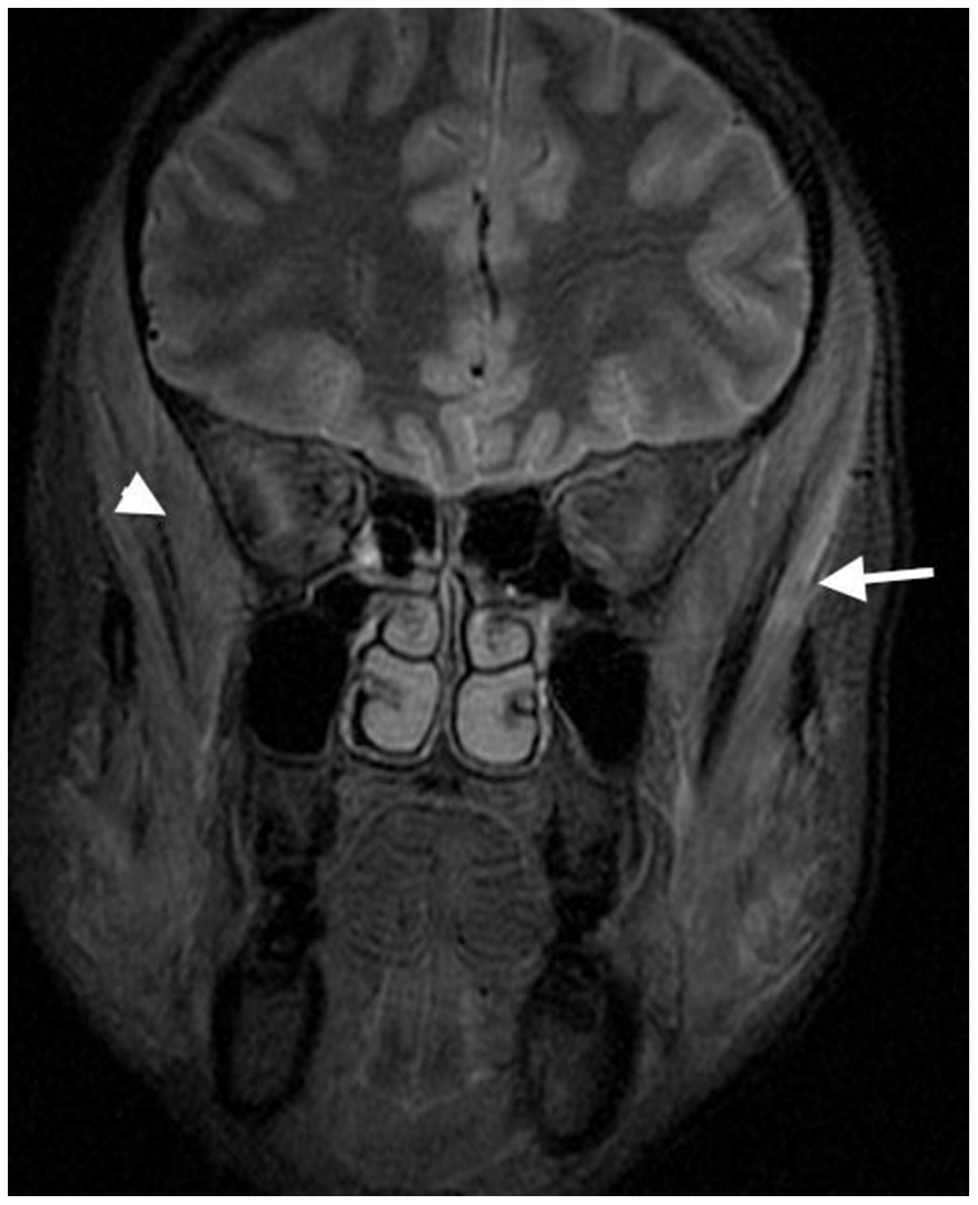
Coronal STIR MRI demonstrating bilateral asymmetrical high signal in deep (arrow head) and superficial (arrow) temporalis muscles (Case 1, Table 3).

**Figure 3:**
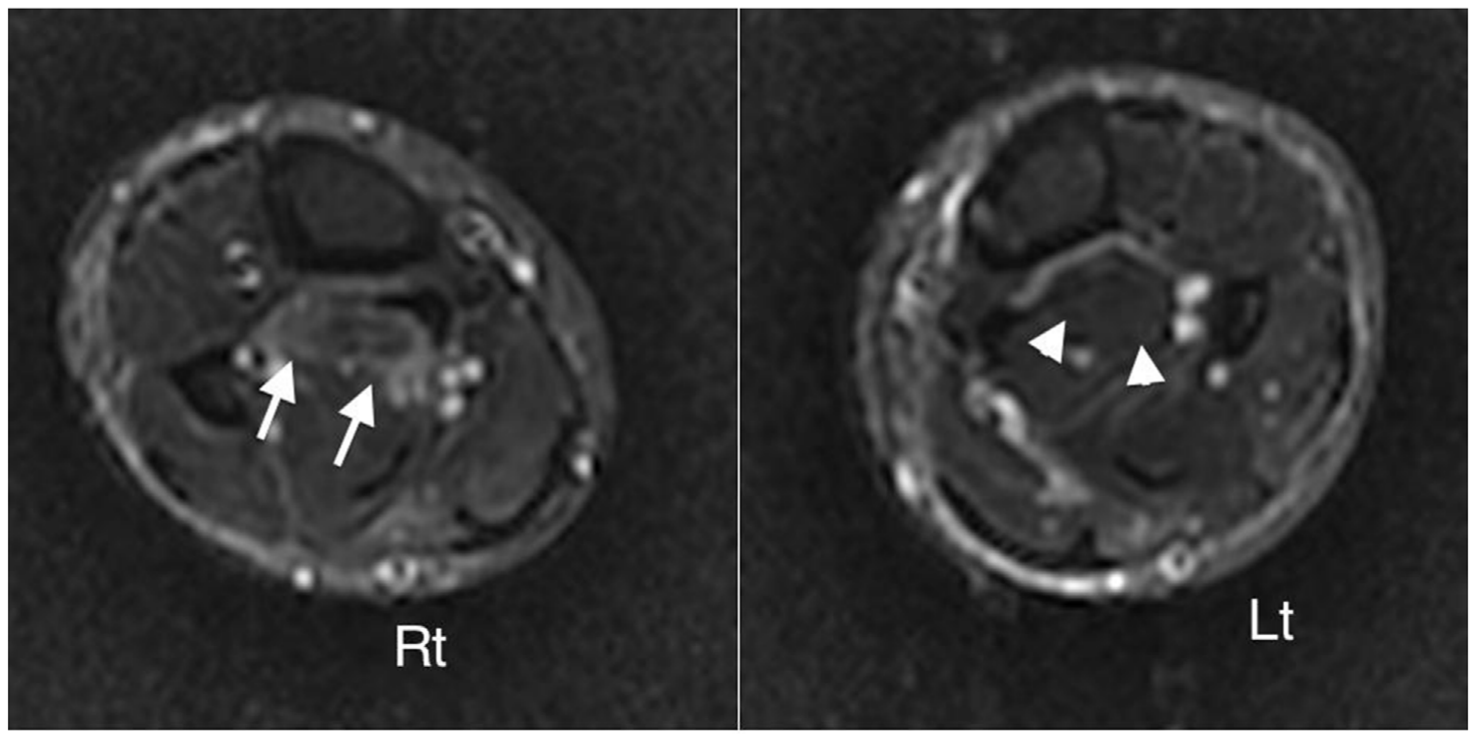
Axial STIR demonstrating heterogenous increased signal right (Rt) tibialis posterior (arrow) compared to non-oedematous muscles (arrowhead) left (Lt) calf (Case 2, Table 3).

Eight patients who had facial swelling and/or myalgia 4–5 weeks after onset of illness underwent MRI examination. Three muscle groups, muscles of mastication, calf, and superficial back muscles demonstrated focal and heterogeneous high signal intensities on STIR consistent with inflammatory edema and myositis. Non-affected muscles demonstrated low signals on STIR. All 8 patients had edema/myositis in the muscles of mastication (superficial temporalis, deep temporalis and masseter muscles) (Figure 2). These were bilateral in 5 and unilateral in 3 patients. Four of these 8 patients had exhibited or reported facial swelling. Four patients had MRI changes in the back muscles and 2 in the calf muscles (Figure 3).

Of the patients who underwent MRI, 2 had both eosinophilia and raised CK, 3 had eosinophilia alone and 3 had only raised CK.

### Muscle Biopsy and *Sarcocystis* spp. Identification

**Figure 4:**
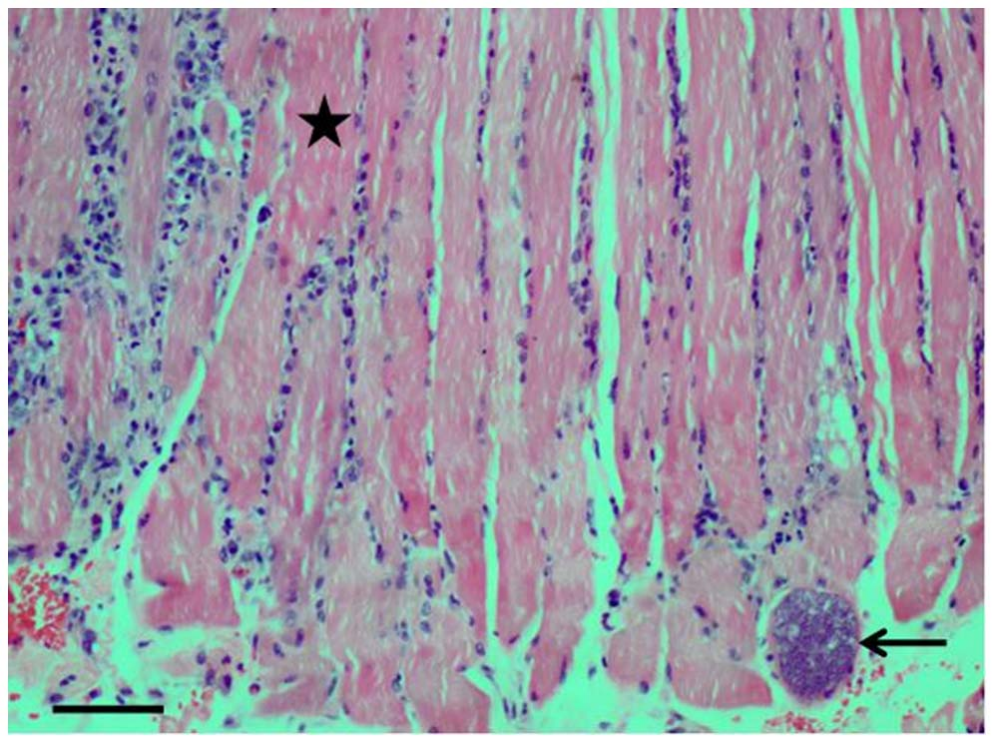
A single sarcocyst (arrow) within a muscle fibre (Case 4, Table 3). Typically there is mild myositis (★) near the sarcocyst. Bar = 40 microns. H&E stains, ×20 objective lens.

Four biopsies were taken from the temporalis (1 case), tibialis posterior (1 case), and gastrocnemius (2 cases) muscles (Table 3). In 3 biopsies (temporalis, tibialis posterior, gastrocnemius), one or more sarcocysts were detected within muscle fibres by light microscopy (Figure 4). A whole sarcocyst was also obtained in a muscle tissue culture preparation from the temporalis muscle. In all 3 biopsies with sarcocysts, there were no inflammatory cells immediately surrounding the infected muscle fibre, but mild to moderate inflammation and focal necrosis was noted in other parts of the muscle. Inflammatory cells were mixed and eosinophils were generally not prominent.

**Table 3:** Clinical features and laboratory findings in 4 muscle-biopsy proven cases.

Case	Symptoms present and duration of symptoms	Number of cycles of relapsing fever	Presence of clinical or MRI evidence of myositis of jaw muscles	Muscle biopsied	Microscopic detection of Sarcocyst	PCR detection of *S. nesbitti*	Peak eosinophil count (normal range 0.02–0.5 10^9^/L)	Peak creatine kinase (normal range 26–192 U/L)
1	Fever, myalgia, headache, arthralgia, cough, vomiting (42 days)	3	Clinical and MRI	Temporalis	Yes	Yes	1.0	40
2	Fever, myalgia, headache, arthralgia, cough, diarrhea (112 days)	2	MRI	Tibialis posterior	Yes	No	0.4	229
3	Fever, myalgia, headache, arthralgia, vomiting (70 days)	3	MRI	Gastrocnemius	No	Yes	2.6	155
4	Fever, myalgia, headache, arthralgia, vomiting (38 days)	3	MRI	Gastrocnemius	Yes	Yes	0.5	1391

We attempted to determine the 18S rDNA sequences from the sarcocysts and/or muscle tissue of 3 biopsies (temporalis, tibialis posterior and gastrocnemius muscles) by polymerase chain reaction (PCR) and direct sequencing [6]. *S. nesbitti* sequences (accession numbers HF544323 and 544324) were confirmed in 2 patients (temporalis and gastronemius muscles). The 18S rDNA gene shared 100% identity with *S. nesbitti* found in the muscle of *Macaca fascicularis* from Yunnan Province, China [9], [10], [11]. The presence of *S. nesbitti* (accession number JX661499.1) was subsequently confirmed by nested polymerase chain reaction and sequencing from the gastrocnemius muscle of the fourth patient (7).

Thus in our series, 4 cases were classified as definite: Three Malaysians and one non-Malaysian. The clinical features and laboratory findings of these 4 cases are shown in Table 3.

### Treatment

As the etiological agent was not determined until late in the course of illness, only 3 patients received medical treatment aside from basic analgesia. Two patients received oral corticosteroids with 1 patient reporting resolution of symptoms soon after commencement of treatment but the other did not report any change.

A third patient received targeted therapy after diagnosis of biopsy confirmed sarcocystosis 10 weeks into illness. With pre-existing renal impairment, clindamycin 600 mg qid po and fansidar (sulfadoxine 500 mg/pyrimethamine 25 mg) 2 tablets/weekly po were prescribed. After 3 days of treatment, the patient reported that pain in the proximal arm muscles and thighs had reduced significantly and proximal arm strength had improved. There was a decrease in CK from 782 to 653 but this had been falling prior to institution of medications. Fansidar was taken for 2 weeks with an interrupted course of clindamycin for 6 weeks. At this point there was no recurrence of myalgia. The patient was subsequently lost to follow-up.

### Exposure Investigation

Water and food contamination were investigated as possible sources of *Sarcocystis* infection. The hotel is located adjacent to a forested area and about 500 m from the beach. The water supply to the hotel came from two sources: chlorinated (treated) water from the mainland and untreated “gravity-feed” water piped down from forested hillsides on the island. Treated water was meant for drinking and preparing food. Untreated water, intended for bathing and washing, was apparently filtered and stored in large outdoor tanks. There was a temporary breakdown in the treated water supply just prior to the retreat. There were reports of the water appearing “cloudy” at the beginning of the retreat and it is possible that untreated water was inadvertently used to prepare food and drinks during the retreat. All 89 symptomatic persons who attended the retreat drank water/beverages prepared in the hotel. As for the 3 persons who did not fall sick, one person drank only Chinese tea prepared with boiled water, while the other two reported drinking ‘mostly’ bottled water. All 92 participants ate meals prepared by the hotel.

*Sarcocystis* spp.were not detected by PCR in any water samples.

## Discussion

This outbreak has previously been described in relation to the molecular identification of *S. nesbitti* as a cause of human muscular sarcocytosis [6], [7]. This report adds to the previous work by providing a comprehensive description of the clinical features of the illness in a large group of affected persons. The focal point of exposure allows for the potential incubation period to be estimated and the temporal changes in both the clinical features and blood investigation results are clearly demonstrated. The important role of MRI is also shown with, for the first time, selected images of the most evident changes. Other aspects of the illness, including treatment options and a possible modification of illness in Malaysians, are also discussed. This report incorporates the clinical, investigative and management aspects of this illness, adding substantially to the previously reported work.

Our findings strongly suggest that muscular sarcocystosis was responsible for the outbreak of acute relapsing febrile myositis in our cohort. Four patients had definite sarcocystosis with sarcocysts or nucleotide sequences demonstrated in the muscles that were shown to be involved by MRI. The other 85 patients had probable muscular sarcocystosis as suggested by the clustered time of onset and recovery from symptoms, overall similarity of symptoms to definite cases, and absence of similar illnesses among patient contacts who did not attend the retreat.

Although sarcocysts have previously been noted as incidental findings in tongue muscles in an autopsy series [5], we believe that their presence in our cases represents a pathological process. Sarcocysts have never been detected in more than 1500 limb muscle biopsies examined at the University of Malaya Medical Centre (Wong KT, unpublished data), thus finding sarcocysts in inflamed skeletal muscles from sites other than the tongue is unlikely to be incidental. In addition, with more than 120 *Sarcocystis* spp. reported in animals [12] it seems unlikely that 3 cases from our cohort could co-incidentally have the same species identified by PCR, with 3 cases also demonstrating sarcocysts of very similar morphology. Although a few previous human studies have shown that febrile myalgia is associated with biopsy-proven, muscular sarcocystosis [3], [4], [13]–[15], this is the first time that *S. nesbitti* has been identified as a cause of symptomatic human muscular sarcocytosis.

Our results suggest that *S. nesbitti* has a very high 97% attack rate. With an identifiable common period of exposure lasting 3 days, the incubation period was determined as most likely to be between 9 and 13 days, but could be up to 28 days. The most common symptoms, fever (94%) and myalgia (91%), were non-specific making initial diagnosis difficult. However, the majority (57.1%) of cases had relapsing fever. Nine patients exhibited facial swelling and an additional 4 had MRI changes consistent with myositis involving the muscles of mastication. Some of the clinical features in the present outbreak appear similar to those described previously. An outbreak involving 6 American military personnel who were believed to be infected in Malaysia, showed that nearly half of them had prolonged fever, myositis and raised liver enzymes [3]. However, there were also reports of bronchospasm, rashes, lymphadenopathy and marked eosinophilia, features not found in our patients. One patient had bitemporal muscle tenderness but facial swelling was not reported. Facial swelling as a striking clinical manifestation has also been reported more recently in a Dutch traveler to Tioman Island, Malaysia [15].

Earlier reports of human muscular sarcocystosis often referred to the disease as an ‘eosinophilic myositis’ [3], [14]. However, we think it important not to exclude this diagnosis on the basis of the eosinophil count since this could be normal early in disease. Normal eosinophil counts were also observed in travellers returning from Tioman Island with possible *Sarcocystis*-like myositis [4]. In fact, in our cohort, lymphocytosis appeared to be as common as eosinophilia throughout the illness.

The apparent clinical differences between our cases and previous reports could be due to a number of reasons. Firstly, it is possible that a pathogenic *Sarcocystis* spp. different from *S. nesbitti* was involved as this is the first time species identification has been successfully performed in human disease. Secondly, the time points at which patients were reviewed appears to affect results as seen in the variations in eosinophil counts and serum CK levels within individual subjects over time. Finally, as the current outbreak occurred within a single community, it is likely that we may have included some patients with milder symptoms who would otherwise not seek medical attention,

Whole body MRI in STIR sequence was helpful to guide the choice of suitable muscle biopsy sites, resulting in a definitive diagnosis in all 4 patients biopsied. This is the highest number of biopsy-proven cases from a single outbreak of symptomatic sarcocystosis, and MRI had an essential role to play. Despite the widespread myalgia, the abnormal MRI changes were confined to 3 distinctive groups comprising of the muscles of mastication, the calves and the back. As discussed, the involvement of the muscles of mastication represents an interesting disease manifestation. In contrast to the utility of MRI, serology performed in a limited number of patients did not appear to be sufficiently sensitive, and this warrants further investigation before diagnostic use in humans can be recommended.

Since clinical manifestations and laboratory investigations may be non-specific, isolated cases of muscular sarcocystosis could be easily missed, and therefore this condition is also likely to be under diagnosed. Nonetheless, prolonged relapsing fever and severe myalgia that lasts several weeks, facial and other muscular swellings, raised LFT, CK, eosinophilia and lymphocytosis could suggest the diagnosis. MRI in STIR sequence, by demonstrating muscles affected by myositis, is a potentially useful tool to guide muscle biopsy to confirm the diagnosis. It cannot be overemphasized that muscle biopsy is most important to confirm the diagnosis and should be done whenever possible. In fact, the definitive diagnosis was only made after muscle biopsy. As our study shows, even in the absence of sarcocysts, the PCR of muscle tissue may be still be useful for diagnosis if clinical suspicion is high.

Interestingly, our study suggests that the only significant risk factor for prolonged disease and moderate/severe myalgia is the patient's nationality. Non-Malaysians were more likely to suffer more prolonged disease and to complain of moderate/severe myalgia than Malaysians. Although perception of severity of pain may be subjective and possibly influenced by cultural values, duration of illness is probably less subjective, suggesting that the observed statistical significance, at least for duration of illness, may indeed be true. The histological demonstration of sarcocysts in 21% of Malaysians along with other reports of incidental findings of sarcocysts in Malaysians suggest that exposure to this organism may not necessarily result in symptomatic infection [5], [16]. However, we speculate that asymptomatic infection could lead to partial immunity which could help explain the shorter duration of symptoms and less severe myalgia experienced by Malaysians in the cohort. It may also explain why all previous reported cases of symptomatic muscular sarcocystosis from Malaysia involved non-Malaysians [3], [4], [13], [15].

There are no conclusive recommendations for the treatment of sarcocystosis [1] although previous reports raise the possibility of clinical improvement with steroids or albendazole but recovery was not rapid [3]. In animals, medications including clindamycin, pyrimethamine and sulphonamides have been suggested as treatment options [17], [18]. There was a previous report of symptomatic improvement and resolution of blood test abnormalities in human sarcocystosis with the use of co-trimoxazole [14]. We elected to treat one patient with prolonged symptoms, as we would a case of toxoplasmosis based on the premise that both organisms are intracellular protozoa. We also elected to utilize medications that have been effective in animals but co-trimoxazole was avoided due to renal insufficiency. Hence the patient received clindamycin and fansidar. It is difficult to attribute the patient's subsequent improvement solely to the medications prescribed but we believe such treatment is worthy of consideration in future cases.

*S. nesbitti* was first described in muscle tissues of *Macaca mulatta* monkeys by Mandour in 1969 [19]. An ultrastructural study of human muscular sarcocystosis in Malaysia has previously shown a similarity to sarcocysts found in *Macaca fascicularis* (Malaysian long tailed macaque) [20]. Phylogenetic analysis suggests that snakes could be a definitive host [10], [19]. More recently, *S. nesbitti* sequences have been detected in the feces of snakes from disparate parts of Malaysia, confirming that snakes may be the definite hosts [7], [11]. As such, *S. nesbitti* is likely to be endemic in the country. We have no evidence for any recent change in ecology of snakes or human/snake interaction to account for the recent cases although further investigations are needed. Reports of 100 foreign persons with an acute muscular *Sarcocystis*-like illness after returning from Tioman Island, Malaysia, between 2011 and 2012 [4] could either suggest a reemerging infection or an increasing recognition of an endemic disease. It is possible that *S. nesbitti* could cause a clinically more apparent disease compared to other *Sarcocystis* spp. that could potentially cause human sarcocystosis. Moreover, it is not known if the severity of clinical disease is dependent on the dose of sporocysts consumed or if a low dose results in largely asymptomatic infection.

Infection in this outbreak is likely due to consumption of water contaminated by snake feces. Almost all the water supply in Malaysia are sourced from streams or rivers, so contamination by snake or other reptile feces may be common. We believe that drinking gravity-feed water contaminated by snake feces or ingestion of uncooked food washed therein, is most likely to be responsible for this outbreak. Water treatment by chlorination may not be able to kill sporocysts [21], thus contaminated piped water from the mainland could still be responsible, albeit less likely as the contaminants are likely to be diluted by a significantly larger water supply. Unsafe water supply, especially in the islands and other remote places, poses potentially serious public health risks that will require urgent investigation and intervention. Travellers should be advised to only drink boiled or bottled water and to avoid uncooked food [21]. There should also be heightened awareness for this infection in patients who have recently lived in or travelled to Southeast Asia.

There were some limitations to our study. Firstly, since it was commenced as an outbreak investigation and the diagnosis was not known until well into the course of the illness, investigations and their timing could not be more uniformly undertaken for all the patients. Secondly, in determining the severity of myalgia the adopted definitions may not be mutually exclusive, and other factors, including cultural, may also have influenced the perception of pain. Finally, due to ethical and other considerations, it was neither possible nor appropriate to perform further muscle biopsies.
